# Supplementary material for: Resolving the full spectrum of human genome variation using Linked-Reads
Source: Genome Res. 2019 Apr;29(4):635–45. doi: 10.1101/gr.234443.118 (PMC6442396; doi:10.1101/gr.234443.118)
Supplement: Supplemental Material [file supp_gr.234443.118_Supplemental_longranger-2.2.zip › longranger-2.2.tar.bz2!/lr2.2-public/lib/stan/cmdstan/stan/doc/api/html/structstan_1_1gm_1_1generate__init__visgen-members.html]

Stan: Member List


|  |  |
| --- | --- |
|  | Stan  2.5.0  probability, sampling & optimization |


- Main Page
- Related Pages
- Namespaces
- Classes
- Files

- Class List
- Class Index
- Class Hierarchy
- Class Members

All Classes Namespaces Files Functions Variables Typedefs Enumerations Enumerator Friends Macros Pages

- stan
- gm
- generate\_init\_visgen

stan::gm::generate\_init\_visgen Member List

This is the complete list of members for stan::gm::generate\_init\_visgen, including all inherited members.

|  |  |  |
| --- | --- | --- |
| function\_args(const std::string &fun\_prefix, const D &x) const | stan::gm::generate\_init\_visgen | inline |
| generate\_buffer\_loop(const std::string &base\_type, const std::string &name, const std::vector< expression > &dims, const expression &dim1=expression(), const expression &dim2=expression(), int indent=2U) const | stan::gm::generate\_init\_visgen | inline |
| generate\_check\_double(const std::string &name, size\_t) const | stan::gm::generate\_init\_visgen | inline |
| generate\_check\_int(const std::string &name, size\_t) const | stan::gm::generate\_init\_visgen | inline |
| generate\_declaration(const std::string &name, const std::string &base\_type, const std::vector< expression > &dims, const expression &type\_arg1=expression(), const expression &type\_arg2=expression()) const | stan::gm::generate\_init\_visgen | inline |
| generate\_dims\_loop\_fwd(const std::vector< expression > &dims, int indent=2U) const | stan::gm::generate\_init\_visgen | inline |
| generate\_indent\_num\_dims(size\_t base\_indent, const std::vector< expression > &dims, const expression &dim1, const expression &dim2) const | stan::gm::generate\_init\_visgen | inline |
| generate\_init\_visgen(std::ostream &o) | stan::gm::generate\_init\_visgen | inline |
| generate\_name\_dims(const std::string name, size\_t num\_dims) const | stan::gm::generate\_init\_visgen | inline |
| generate\_write\_loop(const std::string &write\_method\_name, const std::string &var\_name, const std::vector< expression > &dims) const | stan::gm::generate\_init\_visgen | inline |
| o\_ | stan::gm::visgen |  |
| operator()(nil const &) const | stan::gm::generate\_init\_visgen | inline |
| operator()(int\_var\_decl const &x) const | stan::gm::generate\_init\_visgen | inline |
| operator()(double\_var\_decl const &x) const | stan::gm::generate\_init\_visgen | inline |
| operator()(vector\_var\_decl const &x) const | stan::gm::generate\_init\_visgen | inline |
| operator()(row\_vector\_var\_decl const &x) const | stan::gm::generate\_init\_visgen | inline |
| operator()(matrix\_var\_decl const &x) const | stan::gm::generate\_init\_visgen | inline |
| operator()(unit\_vector\_var\_decl const &x) const | stan::gm::generate\_init\_visgen | inline |
| operator()(simplex\_var\_decl const &x) const | stan::gm::generate\_init\_visgen | inline |
| operator()(ordered\_var\_decl const &x) const | stan::gm::generate\_init\_visgen | inline |
| operator()(positive\_ordered\_var\_decl const &x) const | stan::gm::generate\_init\_visgen | inline |
| operator()(cholesky\_factor\_var\_decl const &x) const | stan::gm::generate\_init\_visgen | inline |
| operator()(cholesky\_corr\_var\_decl const &x) const | stan::gm::generate\_init\_visgen | inline |
| operator()(cov\_matrix\_var\_decl const &x) const | stan::gm::generate\_init\_visgen | inline |
| operator()(corr\_matrix\_var\_decl const &x) const | stan::gm::generate\_init\_visgen | inline |
| result\_type typedef | stan::gm::visgen |  |
| var\_size\_validator\_ | stan::gm::generate\_init\_visgen |  |
| visgen(std::ostream &o) | stan::gm::visgen | inline |


---

[ Stan Home Page ]

*© 2011–2014,
Stan Development Team.*
